# The Microbiota–Human Health Axis

**DOI:** 10.3390/microorganisms13040948

**Published:** 2025-04-20

**Authors:** Harrie Toms John, Treesa Clare Thomas, Ezenwa Collins Chukwuebuka, Ali Bacar Ali, Reggani Anass, Yididiya Yilma Tefera, Bency Babu, Nicoleta Negrut, Anca Ferician, Paula Marian

**Affiliations:** 1 Department of Intensive Care, Epsom and St. Helier University Hospitals NHS Trust, Wrythe Ln, Sutton SM5 1AA, UK; 2 Faculty of Medicine and Pharmacy, University of Oradea, Piaţa 1 Decembrie 10, 410068 Oradea, Romania; treesaclarethomas2902@gmail.com (T.C.T.); cascollins@yahoo.com (E.C.C.); alibacar.ali@hotmail.com (A.B.A.); anassreg99@gmail.com (R.A.); 3 Lahti Health and Social Services Centre, Harjukatu 48, 15100 Lahti, Finland; tefera.yididiya@gmail.com; 4 Department of General Internal Medicine, Northampton General Hospital, NHS Trust, Northampton NN1 5BD, UK; bency.babu@nhs.net; 5 Doctoral School of Biomedical Sciences, Faculty of Medicine and Pharmacy, University of Oradea, 410087 Oradea, Romania; 6 Department of Psycho-Neuroscience and Recovery, Faculty of Medicine and Pharmacy, University of Oradea, 410073 Oradea, Romania; 7 Department of Medical Disciplines, Faculty of Medicine and Pharmacy, University of Oradea, 410073 Oradea, Romania; anca.moza@yahoo.com (A.F.); paula.marian85@gmail.com (P.M.)

**Keywords:** microbiota, microbiome transplantation, dysbiosis, probiotics

## Abstract

Trillions of microorganisms play a pivotal role in maintaining health and preventing disease in humans. Their presence influences daily life, habits, energy levels, and pathologies. The present narrative review synthesized recent studies of microbial diversity across organ systems. The composition of the microbiota regulates the intestinal barrier, modulates the immune response, influences metabolism, and produces essential compounds such as short-chain fatty acids and neurotransmitters. Dysbiosis is associated with numerous pathologies, including metabolic, autoimmune, neurodegenerative, and cardiovascular diseases. The microbiota is key to maintaining physiological balance and reducing disease risk. Therapeutic interventions, such as probiotics, prebiotics, postbiotics, and microbiome transplantation, offer promising perspectives in restoring microbial homeostasis and preventing chronic diseases.

## 1. Introduction

The human microbiota, a complex and dynamic ecosystem, consists of approximately 100 trillion microorganisms, three times more than human cells, including bacteria, viruses, fungi, archaea, and unicellular eukaryotes. It weighs around 1.1 kg and occupies 1.4 L, with the highest density found in the intestine [1,2]. These microorganisms interact with the host, profoundly influencing physiological homeostasis and immune mechanisms [3].

Recent research highlights the significance of the microbiota across various aspects of human health. The proper functioning of the host organism depends on the microbiota, which is involved in processes such as metabolism, immune responses, digestion, intestinal permeability, bone growth, and development, highlighting its importance in maintaining health and preventing diseases [4,5,6,7,8]. This narrative review explores these complex relationships to provide an updated perspective on the therapeutic potential of microbiota interventions, including probiotics, prebiotics, and fecal microbiota transplantation.

## 2. Materials and Methods

This narrative review analyzed the medical literature regarding the role of the microbiota in human health. Relevant references were identified from scientific databases, including theNational Center for Biotechnology Information (NCBI, Bethesda, MD, USA), Centers for Disease Control and Prevention (CDC, Atlanta, GA, USA), Scientific Electronic Library Online (SciELO, São Paulo, Brazil), Scopus (Elsevier, Amsterdam, Netherlands), Web of Science (Clarivate Analytics, Philadelphia, PA, USA), and the Cochrane Database (Cochrane, London, UK). A literature search was performed for publications from 2014 to 2024 using the terms “microbiota” or “microbiome”, in combination with “digestive system”, “metabolism”, “immune system”, “integumentary system”, “respiratory system”, “urinary system”, “reproductive system”, “central nervous system”, “cardiovascular system”, and “endocrine system”.

The selection included peer-reviewed articles, reviews, clinical trials, observational studies, and meta-analyses written in English, Italian, or Romanian, focusing on the impact of the microbiota on human health and disease. The studies were selected based on their scientific relevance and contribution to the field. Articles lacking full-text availability, insufficient data to support conclusions, or focused on non-human microbiota were excluded. A total of 46,903 records were initially identified through the keyword search. After screening and eligibility assessment, 129 publications were ultimately included in the final analysis.

## 3. The Multisystem Impact of Microbiota

### 3.1. Digestive System and Metabolism

The human digestive system harbors a vast and complex community of microorganisms known as the microbiota. These microbes are essential for overall health, aiding digestion, supporting immune function, and maintaining metabolic balance. Predominantly composed of bacteria, these microbial communities support digestive health by breaking down indigestible food and synthesizing essential vitamins and short-chain fatty acids (SCFAs). Due to microbial activity, complex polysaccharides are converted into absorbable substrates, with these microorganisms coexisting in the digestive tract without typically causing clinical disorders. The gut flora also plays a crucial role in regulating the immune system by protecting against pathogens and modulating immune responses [9]. Numerous recent studies highlight the role of the gut microbiota in behavioral modulation through the gut–brain axis. Maintaining microbial equilibrium is important to prevent inflammation caused by increased intestinal lining permeability, which can trigger heightened immune reactions.

**Figure 1:**
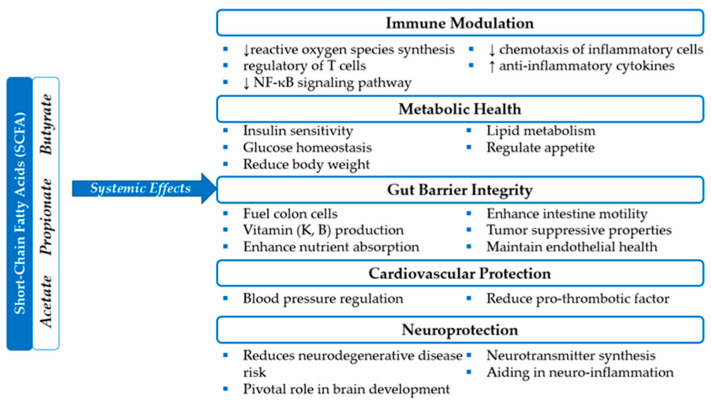
The role of SCFA in systemic health. SCFA—short-chain fatty acids; NF-κB—Nuclear Factor kappa-light-chain-enhancer of activated B cells.

Fermentation of complex polysaccharides, such as dietary fibers, which human enzymes cannot digest, is one of the primary functions of gut microbes [10]. SCFAs such as butyrate, propionate, and acetate are produced in the large intestine and play a critical role in metabolic homeostasis. These compounds serve as primary energy sources for colonocytes, regulate intestinal motility, protect the intestinal mucosal layer, improve nutrient absorption, and modulate systemic inflammatory responses, Figure 1 [10,11,12]. Butyrate, produced by bacteria such as *Faecalibacterium prausnitzii*, *Eubacterium rectale*, and *Roseburia* spp., has been shown to have anti-inflammatory and antitumor properties, contributing to the maintenance of intestinal mucosal health and reducing the risk of conditions such as leaky gut syndrome [12,13]. Additionally, studies indicate that butyrate stimulates the production of mucin and antimicrobial peptides, which protect the intestinal mucosa from infectious agents [12]. The degradation of glycoproteins in the intestinal mucus, carried out by bacteria such as *Akkermansia muciniphila*, *Bacteroides fragilis*, and *Ruminococcus gnavus*, provides essential nutrients for the gut microbiota and contributes to maintaining the integrity of the intestinal barrier [14,15,16].

The gut microbiota is involved in protein digestion processes (dietary, host-derived, or produced by microorganisms). Species include *Clostridium* spp., *Bacteroides* spp., *Proteus* spp., and *Peptostreptococcus* spp. which break down undigested proteins into secondary metabolites, such as amino acids and small peptides, which are further transformed, through fermentation and deamination processes, into end products like SCFAs, ammonia, biogenic amines, phenols, and indoles [17]. These metabolites can either be eliminated by the host or used in specific biochemical reactions to synthesize other compounds.

A high-protein diet, as well as the type of protein ingested, influences the composition of the gut microbiota, leading to variations in the concentrations of different bacterial families and major phyla (*Actinobacteria*, *Bacteroidetes*, *Firmicutes*, *Proteobacteria*, *Tenericutes*, and *Spirochaetes*). These microbial shifts may contribute to the onset and progression of pathological conditions such as inflammatory bowel diseases, colorectal cancer, and metabolic disorders (e.g., type 2 diabetes) [17].

The microbiota also plays a key role in xenobiotic metabolism, either by degrading compounds into inactive metabolites (as seen with digoxin in individuals colonized with *Eggerthella lenta*) or by activating them into prodrugs, such as humimycin antibiotics synthesized by *Rhodococcus* spp. or the transformation of soy-derived isoflavones into equol, an estrogen-like substance. Additionally, the microbiota can contribute to synthesizing toxic metabolites, especially following their re-entry into the intestine through the enterohepatic circulation of compounds previously detoxified by the host. Certain bacterial species, such as *Lactobacillus* spp. and *Bifidobacterium* spp., bind aflatoxins and heavy metals (mercury, cadmium, lead), facilitating their elimination, while *Akkermansia muciniphila*, by supporting the mucus layer, can limit xenobiotic absorption, acting as a physical barrier [18].

The gut microbiota is essential for the synthesis of B-complex vitamins such as thiamine (B1), riboflavin (B2), niacin (B3), pantothenic acid (B5), pyridoxine (B6), biotin (B7), folate (B9), cobalamin (B12), and K (K2) which collectively support critical metabolic processes including carbohydrate and fat metabolism, DNA synthesis, neurotransmitter production, red blood cell formation, blood clotting, bone health, and maintaining vascular integrity. Vitamin synthesis varies by age. In infants, species such as *Escherichia coli*, *Bacteroides* spp., *Veillonella parvula*, *Prevotella copri*, *Phocaeicola dorei*, *Bifidobacterium breve*, *Bifidobacterium bifidum*, and *Faecalibacterium prausnitzii* are involved in the production of B-complex vitamins, while *Escherichia coli*, *Bacteroides* spp., *Veillonella parvula*, and *Phocaeicola dorei* contribute to the synthesis of vitamin K [19]. In adults, the synthesis of B-complex vitamins is supported by *Bacteroides* spp., *Eubacterium rectale*, *Phocaeicola dorei*, *Bifidobacterium adolescentis*, *Faecalibacterium prausnitzii*, *Ruminococcus gnavus*, and *Prevotella copri*, whereas vitamin K production involves *Bacteroides* spp., *Eubacterium rectale*, and *Phocaeicola dorei* [19]. In elderly individuals, species such as *Escherichia coli*, *Bacteroides* spp., *Klebsiella pneumoniae*, *Prevotella copri*, *Phocaeicola dorei*, *Ruminococcus gnavus*, and *Alistipes putredinis* contribute to B-complex vitamin synthesis, while *Escherichia coli*, *Bacteroides* spp., *Klebsiella pneumoniae*, and *Phocaeicola dorei* are responsible for vitamin K production [19]. These microbial dynamics highlight the age-dependent role of the gut microbiota in supporting metabolic and physiological functions. Any disruption in this balance can negatively impact metabolic health, as the symbiosis between the gut microbiome and humans is vital for optimal digestive health [20]. This also underscores the significance of a healthy and diverse microbiota for the overall well-being of a human being.

The gut microbiome’s impact extends beyond the digestive system, profoundly affecting the human body’s metabolic regulation. One of these roles is lipid metabolism. Processes such as deconjugation and dihydroxylation, carried out by species of the human microbiota, transform primary bile acids (BAs) into secondary BA, which are essential for emulsifying, breaking down, and absorbing lipids [21,22]. Cholesterol metabolism is regulated through the interaction between the gut microbiota and BA [23]. Bacteria such as *Eubacterium coprostanoligenes*, *Bifidobacterium* spp., and *Lactobacillus* spp. convert cholesterol into coprostanol, a less absorbable compound excreted in feces [22]. Trimethylamine N-oxide (TMAO) disrupts cholesterol and lipoprotein metabolism, contributing to vascular inflammation, plaque formation, and an increased risk of cardiovascular disease and diabetes [24,25,26,27,28]. TMAO is produced in the liver from trimethylamine (TMA), which is generated in the gut by bacterial species such as *Firmicutes* spp., *Proteobacteria* spp., *Clostridium* spp., *Desulfovibrio* spp., and *Anaerococcus* spp. through dietary choline and carnitine metabolism, primarily sourced from high-fat foods like red meat and eggs [28]. *Bacteroides* spp. and *Bifidobacterium* spp. metabolize dietary sphingolipids and simple sphingoid bases from the diet, improving intestinal barrier function, reducing exposure to bacterial toxins, and supporting immunity, with a potential role in regulating metabolic health and neurodevelopment [22]. Additionally, bacteria such as *Bacteroides* spp. and *Lactobacillus* spp. modulate the production of eicosanoids (prostaglandins and leukotrienes) by metabolizing essential fatty acids (omega-3 and omega-6), influencing inflammatory processes and overall metabolic balance [22].

The gut–brain axis, a bidirectional communication route between the digestive and central nervous systems, highlights the microbiota’s role in regulating appetite, satiety, and energy production. Gut hormones, such as glucagon-like peptide-1 (GLP-1) and PYY, are released in response to microbial metabolites, including SCFAs. These hormones are critical in maintaining appetite, glucose balance, and insulin sensitivity [24].

Studies have shown that metabolic disorders such as heart disease, hypertension, obesity, insulin resistance, gastrointestinal disorders, and type 2 diabetes, as well as an increased risk of cancers, are associated with dysbiosis [25,26]. Obese individuals show a specific microbial signature, characterized by reduced microbial diversity and an elevated *Firmicutes*/*Bacteroidetes* ratio, associated with a higher capability to harvest energy from the diet [27]. A 2022 study by Zhou et al. stated a decline in gut microbial diversity and deterioration of butyrate-producing bacteria in individuals with type 2 diabetes [25].

Certain gut microbes play a direct role in colorectal carcinogenesis. *Fusobacterium nucleatum* is enriched in colorectal cancer (CRC) tissues and promotes tumor progression via its adhesins Fusobacterium adhesin A (FadA) and Fusobacterial apoptosis protein 2 (Fap2). FadA binds to the epithelial adhesion protein E-cadherin, activating the β-catenin signaling pathway and promoting cellular proliferation. Fap2 impairs antitumor immunity by binding to TIGIT (T cell immunoreceptor with Ig and ITIM domains) on natural killer cells, allowing cancer cells to evade immune detection. *Fusobacterium nucleatum* has also been detected in primary tumors and liver metastases, suggesting its role in cancer dissemination. Similarly, enterotoxigenic *Bacteroides fragilis* (ETBF) produces B. fragilis toxin (BFT), a metalloprotease that disrupts epithelial barrier integrity and induces interleukin-17 (IL-17)-mediated inflammation, enhancing tumor development. In mouse models, ETBF colonization leads to rapid colonic tumor formation in genetically susceptible hosts [29].

The gut microbiome can be influenced by various factors, with diet playing a central role. Diets rich in plant-based fiber are generally associated with an increased presence of beneficial bacteria, such as *Bifidobacteria* and *Lactobacillus*. In contrast, high-fat and high-sugar diets tend to reduce microbial diversity and promote the growth of pro-inflammatory species, potentially contributing to metabolic imbalance [30].

Prebiotics and probiotics have emerged as promising advancements towards improving digestive health. Prebiotics are non-digestible food compounds, such as inulin, which nourish beneficial microbes and foster their growth, and probiotics are live microorganisms found in fermented foods and supplements that help replenish the microbial balance in the gut. The introduction of both has shown a positive effect on overall human well-being by potentially reducing metabolic diseases by decreasing inflammation, enhancing gut barrier functions, and modulating glucose uptake.

In addition to dietary interventions, fecal microbiota transplantation (FMT) is an approved treatment for recurrent *Clostridioides difficile* infection, while its use in inflammatory bowel disease (ulcerative colitis and Crohn’s disease), irritable bowel syndrome, severe post-antibiotic gut dysbiosis, metabolic disorders (obesity and type 2 diabetes), microbiota-associated neurological disorders (autism, Parkinson’s disease, and multiple sclerosis), and graft-versus-host disease following hematopoietic stem cell transplantation remains investigational or off-label [31,32,33].

### 3.2. Integumentary System

Skin, a multidimensional organ, is vital in preserving physical harmony by defending against external threats, regulating fluid balance, controlling temperature, sensing stimuli, and combating pathogens. Humans represent a minority compared to the microbes inhabiting our skin, as shown in Table 1 [34,35].

The first contact begins with vaginal birth, while c-section offers a more sterile environment [36]. After that, different relationships between the human skin and microorganisms develop, encompassing mutualistic, communal, saprophytic, and parasitic interactions. The skin microbiome varies significantly across different body regions and changes with genome, gender, age, and environmental factors such as living conditions, antibiotic use, sun exposure, and diet.

The stratum corneum, consisting of corneocytes surrounded by lipid layers, creates a resilient physical barrier that prevents water loss and protects against external threats [37,38]. Lower temperature, fatty acid production, and sweat gland secretion of lactic acid maintain acidity, supporting beneficial bacteria while inhibiting pathogenic strains like *Staphylococcus aureus* [39,40]. Most skin areas are dry, which is generally an unfavorable environment for bacterial growth. The continuous shedding of epithelial cells in the stratum corneum renews the skin surface and removes adhering microorganisms [41]. The skin’s defense also includes a chemical barrier of antimicrobial peptides, proteases, cytokines, and chemokines, which can directly inhibit microbial growth or activate immune responses [42].

Age significantly affects the diversity and composition of skin microbiota. Studies suggest that adults have higher microbial diversity than adolescents and the elderly. The increased microbial diversity in adults can be explained by the accumulation of several factors, such as hormonal stabilization, cumulative exposure to a wide range of environments and external factors throughout life, lower use of antibiotics compared to adolescents, and better skin barrier integrity than in older people. Furthermore, the daily activities and diversity of human interactions in which adults are involved, compared to older people, may contribute to the maintenance and diversification of the skin microbiome. Skin moisture retention and sebum production are influenced not only by age but also by gender. Lipophilic bacteria like *Propionibacterium* increase in abundance during adolescence, peaking in the third decade of life, which correlates with sebum levels [43].

Physiological variances in hormone metabolism, perspiration rate, and skin surface pH between males and females contribute to gender variations in skin environments [44]. While previous research highlighted significant alpha diversity differences between genders at the palm only, current studies identify these distinctions across all body sites, particularly the glabella [44]. The specific reasons for gender variations in the glabella remain theoretical, but differences in facial cosmetic usage within the genders could potentially play a role.

Urban and rural environments differ significantly, as do the lifestyles of their inhabitants. For instance, rural adults and the elderly are more likely to engage in agricultural work, while urbanites typically have indoor occupations. These lifestyle disparities can affect skin conditions and the microbial communities present there. Indoor microbiomes predominantly originate from humans, whereas outdoor microbial sources include soil, water, animals, atmosphere, and plant-associated microbes, which can influence the composition of an outdoor worker’s skin microbiome [45,46].

The primary commensal microbes on the skin are *Staphylococcus epidermidis* (*S. epidermidis*), *Cutibacterium acnes* (*C. acnes*), formerly *Propionibacterium acne*, and *Corynebacterium*. Unfortunately, our knowledge about other bacterial species on the skin is limited due to their low quantity and typically harmless nature [47]. The successful colonization of human skin by *S. epidermidis* is due to its commensal lifestyle, which prioritizes traits favoring persistence without harming the host. With low cytotoxicity and the ability to evade host defenses, *S. epidermidis* minimizes immune responses, resembling a mutual non-aggression pact. Certain characteristics suggest a symbiotic relationship, aiding the defense against transient pathogens. However, in compromised host immunity scenarios such as injury or surgery, *S. epidermidis* may exhibit opportunistic pathogenic behavior [48]. A reduced presence of commensal strains is often linked with an increased risk of pathogenic colonization [49]. Studies show that coagulase-negative *S. epidermidis* produces antimicrobial peptides (AMPs) that protect against colonization by the pathogenic *Staphylococcus aureus* [50]. *C. acnes* exhibits both non-pathogenic and pathogenic characteristics. It shares some properties with the ‘Generally Recognized as Safe’ (GRAS) status microbiome [51]. The relationship between the host and *C. acnes* consists of both mutualistic and parasitic elements, depending on the host’s tolerance, genetics, hormones, age, and the anatomical site of infection [49]. The sebaceous glands of the scalp, face, chest, and back produce large amounts of sebum, where the lipophilic anaerobe *C. acnes* predominates [52].

*Coryneform* spp. bacteria include several species, with *Corynebacterium* (*C*) being the most common. *Corynebacterium* is a Gram-positive pleomorphic rod divided into diphtheria and non-diphtheria species (diphtheroids). They are harmless bacteria that inhabit moist areas such as armpits and groins [53]. Beyond their commensal role, where they compete for nutrients and space, certain *Corynebacterium* species contribute to colonization resistance by producing bacteriocins (*C. jeikeium*), inhibiting *Staphylococcus aureus* virulence factors (*C. striatum*), or generating antimicrobial fatty acids (*C. accolens*). Among the more than 130 recognized species, *C. jeikeium*, *C. striatum*, *C. amycolatum*, *C. minutissimum*, *C. kroppenstedtii*, and *C. xerosis* can become opportunistic pathogens, particularly in immunocompromised patients. *Corynebacterium* has become a clinical concern due to its increasing antibiotic resistance, especially in nosocomial infections [54].

The fungal variety within the skin’s microbiota is limited, with the most predominant species being *Malassezia* spp. Studies estimate that *Malassezia* spp. account for 60-90% of the total fungal population on human skin [55]. Due to their inability to synthesize lipids, they are primarily found in sebaceous skin areas such as the scalp, face, back, and chest. Other fungal residents of the skin, like *Candida albicans* and dermatophytes, are only distantly related to *Malassezia*. Despite over a century of known associations with various diseases, the role of *Malassezia* in health and disease remains debated. Culture-based assessments indicate that *Candida* spp. infrequently inhabit human skin but may cause clinical infections, particularly in individuals with compromised immune systems, diabetes, or following antibiotic therapy. Another additional fungal genus reported to be present in the skin microbiota is *Debaryomyces hansenii*. [56].

Viruses inhabit healthy skin and are often considered pathogenic agents. This was evidenced by the asymptomatic presence of β- and γ-human papillomaviruses and viruses from the *Polyomavirus* genus [49,57]. *Merkel cell polyomavirus* (MCPyV), originally isolated from an aggressive neuroendocrine skin tumor known as Merkel cell carcinoma (MCC), was discovered on the skin surface of most healthy individuals, as well as on normal or pathological skin in patients with benign or non-MCC malignant skin conditions [57]. Viruses can positively impact homeostasis by regulating microbial populations and controlling overgrowth [58].

Skin is the body’s largest organ, extending from head to toe. The roles of the cutaneous microbiota are multiple. The commensal microorganisms at this level form a natural protective barrier against skin colonization by pathogenic microorganisms, competing for space and nutrients and synthesizing antibacterial molecules (natural antibiotics, free fatty acids, AMPc) that inhibit pathogens [35]. The microbiota stimulates the immune system by maintaining the local pro- and anti-inflammatory balance and activating local immune cells. These microorganisms regulate the skin’s pH, maintain the integrity of the stratum corneum, control local inflammatory processes, and preserve the skin’s homeostasis [35]. Some microorganisms can stimulate cellular regeneration processes, promoting the repair of skin lesions. A balanced cutaneous microbiota reduces the risk of skin conditions such as acne, atopic dermatitis, psoriasis, chronic wounds, and bacterial or fungal local infections [35]. Lastly, the microbiota helps the skin adapt to various environmental conditions, such as temperature or humidity variations and exposure to chemical agents.

Cutaneous dysbiosis has been associated with atopic dermatitis, acne, and microbial imbalances responsible for unpleasant body odor. In this context, skin microbiome transplantation (SMT), an emerging strategy, aims to restore the skin microbiota by applying selected beneficial microorganisms or directly transferring microbiota from healthy donors. Preclinical studies support that therapeutic colonization with *Staphylococcus epidermidis* and *Staphylococcus hominis* can reduce *Staphylococcus aureus* presence in patients with atopic dermatitis, with *Roseomonas mucosa* improves its evolution, while non-inflammatory *Cutibacterium acnes* strains may help regulate the microbiome in acne vulgaris [59,60]. Additionally, axillary microbiota manipulation has shown a potential to reduce body odor by replacing odor-causing bacteria with non-odorous species [60,61].

Although SMT shows therapeutic promise, research is still in its early stages, and aspects regarding this intervention’s stability, efficacy, and safety require further investigation. Integrating this approach into clinical practice could open new avenues for personalized dermatological therapy.

### 3.3. Respiratory System

The healthy lung was traditionally considered a sterile organ, particularly the distant alveoli [62]. We now know that it supports a rich and varied microbiota. A large part of these microbial communities, which are made up of bacteria, viruses, and fungi, is responsible for maintaining the normal physiological activities of the respiratory system [63]. Previously, numerous studies have been performed on the impact of the gut microbiota on human health and disease; however, in recent years, the impact of the microbiome on lung health and disease has been increasingly discussed [64,65]. Only recently have the functional mechanisms and consequences of microbiomes on lung health been explained. The respiratory tract microbiota has multiple roles in maintaining respiratory health, the most important being to serve as a gatekeeper to prevent respiratory infections from colonizing the system [66,67].

#### 3.3.1. Upper Respiratory Tract Microbiota

The upper respiratory tract provides a variety of micro-niches, shaped by local anatomy, constant exposure to the external environment, and immune activity. Microbial colonization begins immediately after birth, influenced by the mode of delivery and type of feeding, with breastfeeding promoting dominant species such as *Dolosigranulum* and *Corynebacterium*, while formula feeding favors *S. aureus* [68]. As maturation progresses, the microbiome becomes more diverse in adults, with *Actinobacteria*, *Firmicutes*, and *Bacteroidetes* predominating, while in the elderly, there is a transition toward microbial communities resembling those of the oropharynx, possibly as a result of immunosenescence and reduced local biodiversity [68].

In addition to bacteria, this microbiome includes other microorganisms such as *Thaumarchaeota* and methanogenic *Euryarchaeota* archaea, as well as various fungal and viral species, although these remain less well studied [68]. Furthermore, the respiratory mucosa secretes antimicrobial substances such as lysozyme, lactoferrin, defensins, and reactive oxygen species like nitric oxide, which contribute to pathogen control and the maintenance of mucociliary clearance. Processes such as detecting bacterial molecules by specific taste and olfactory receptors can activate rapid immune responses, even before bacteria reach a significant pathogenic load.

Exposure to external factors, such as smoking and inhaled air quality, influences the microbiome at this level, increasing its diversity but reducing its stability, with a tendency to favor potentially pathogenic bacteria. In contrast, interventions such as nasal lavage with saline solutions and the administration of probiotics have proven to be effective and gentle methods for supporting microbiome balance and eliminating inflammatory mediators and pollutants.

#### 3.3.2. Lower Respiratory Tract Microbiota

The lungs are continually exposed to germs suspended in the air and upper airways [69]. Since over 80% of the bacteria that inhabit the human body are undetectable by conventional culture-based methods, it is challenging to demonstrate the presence of the microbiome in the lungs. However, recent advances in sequencing technologies like amplicon have increased our comprehension of the lung microbiome [62,70,71].

A wide variety of microorganisms colonize the lung’s mucosal tissue. The lungs and airways are colonized by microorganisms from the oral cavity and environment [72]. The most common class of microorganisms in the lungs of healthy people are *Prevotella*, *Streptococcus*, *Veillonella*, *Neisseria*, *Haemophilus*, and *Fusobacterium* [73,74,75]. This diverse microbiota forms a complex and heterogeneous ecosystem that supports several key physiological processes [5,62,76]. Most flora is aerobic or facultatively anaerobic, except for *Clostridium*, *Veillonella*, and *Porphyromonas*, specialized anaerobes [63].

The lung microbiota plays essential roles in maintaining immune homeostasis, modulating inflammatory responses, and protecting against respiratory pathogens. The host’s immune system is modulated predominantly by the lung bacteria [77]. The lining of the lungs is covered in a thin layer of mucus with bacteriostatic properties, which offers microorganisms a low-nutrient habitat. The lung microenvironment is characterized by high immunological “tolerance”, which is primarily maintained by subpopulations of alveolar macrophages (AMs) and dendritic cells (DCs) [66]. These cells demonstrate their immunoregulatory capabilities by triggering the production of regulatory T cells (Treg) and releasing prostaglandin E2 (PGE2), tumor growth factor-beta (TGF-β), and interleukin-10 (IL-10). There is growing evidence that the lung microbiota plays a crucial role in fostering lung immunological tolerance by influencing resident immune cells [76,77]. These microorganisms help the lung react to dangerous infections correctly, preventing excess inflammatory reactions to benign antigens [72,78]. Microbial compounds and immune cell pattern recognition receptors (PRRs) interact to accomplish this immunological control [66]. These interactions prevent excessive immune responses and regulate inflammatory pathways. Additionally, immunoglobulin A (IgA), which covers mucosal surfaces and maintains immunological homeostasis by neutralizing pathogens before they can infiltrate deeper into lung tissues, is partly produced by the microbiota [79].

The lung microbiota supports the respiratory system’s physical defenses, such as the mucosal barrier and mucociliary clearance. Inhaled particles and microorganisms are trapped by a mucus layer that protects the epithelial cells lining the airways. The synthesis and composition of this mucus are influenced by microbial communities, which maximize its ability to capture dangerous particles while permitting effective gas exchange. Furthermore, the microbiota facilitates the movement of cilia, which are tiny hair-like projections on the surface of airway epithelial cells that aid in removing mucus and debris from the lungs [63,67,80].

The host’s mucosal barriers and microbial communities must balance to avoid infections and preserve lung health. Because the microbiota impacts mucociliary function, pathogens are effectively eliminated, lowering the risk of respiratory infections and enhancing the respiratory system’s overall resistance [67].

Despite the structural differences between the gut and lungs, possible anatomic communications and intricate networks involving their respective bacteria have supported the possibility of a gut–lung axis. According to the gut–lung axis theory, changes in the gut microbiome’s components brought on by illness, food, or medications are associated with altered immune responses and airway homeostasis [64,77,81,82,83]. Even though our understanding of the gut–lung axis is still developing, new research suggests lung disorders may be treated by modifying the gut microbiota [77]. The gut microbiota produces microbial metabolites, like SCFAs, which can enter the circulation or lymphatic system and reach the lungs, influencing immunological responses and supporting lung homeostasis [84]. Numerous studies show that the gut and lung microbiota are important for maintaining pulmonary health, and as such, they could be targets for nutrition-based preventive strategies against lung disease in older populations. This is because communication along the gut–lung axis becomes more pronounced with age or in elderly patients [82].

### 3.4. Urinary and Reproductive Systems

The urinary microbiota is the microbial community in the urinary system within the bladder or in bladder-obtained urine [85,86]. Previously, urine was said to be sterile, but bacterial communities have been discovered in the bladder, which has a potential role in urinary health [85,87,88]. Recent studies documented a wide spectrum of microbiota associated with healthy, culture-negative urine [85,86]. Many of these urinary microbial communities are like those seen in the human vagina, colon, and skin [89].

The five predominant bacteria in the urinary system of healthy males were *Streptococcus*, *Lactobacillus*, *Prevotella*, *Corynebacterium*, and *Pseudomonas*, and these were also seen in the urethral swab, although at varying degrees [86,89,90]. The microbiota at the penile sulcus is predominantly *Pseudomonadaceae* spp., which is rare in urine, whereas *Sneathia* spp. and *Lactobacillus* spp., which are common in urine, are rare in the sulcus of uncircumcised men [89]. It was observed that some of the penile microbiota, such as *Pseudomonadaceae* and *Oxalobactericeae*, were the same irrespective of circumcision status, but before circumcision, the putative anaerobic bacterial microbiota belonging to the families of *Clostridiales* and *Prevotellaceae* were more prevalent [90].

The five most predominant urinary microbiota in healthy females were *Lactobacillus*, *Corynebacterium*, *Gardnerella*, *Prevotella*, and *Bacillus* [87]. For the female reproductive system, many of the same urinary microbiota were cultured from the vaginal specimen, such as the *Lactobacillus*, *Gardnerella*, *Bifidobacterium*, *Enterococcus*, *Actinomyces*, *Prevotella*, and *Atopobium* [87]. This shows that both the urinary and vaginal tracts may have a common urogenital microbial community, although another study reported that the urethra and bladder may have distinct microbial communities from the vagina [90]. A healthy vaginal microbial community, however, consists of *Lactobacillus*, and it seems to play a key role in preventing several urogenital diseases by lowering the environmental pH through lactic acid production [86,87,89,90,91,92,93]. A pH below 4.5 is critical for the ecological balance of the vaginal microbiome, primarily sustained by lactic acid produced by *Lactobacilli* from epithelial glycogen stores under estrogenic stimulation. It was also observed that most microbial communities are commonly shared by men and women, except *Lactobacillus crispatus*, found only in healthy women [91]. In summary, the most common microbiota detected in males’ urinary and reproductive systems was *Streptococcus*, while in females, it was *Lactobacillus* [86,87,88,89,91,92,93].

There are several ways in which these microbial communities impact the urinary and reproductive systems. The abundance of vaginal lactobacilli prevents urinary tract infections (UTI) as it has been shown that the depletion of *Lactobacillus* in the microbiota is associated with UTI risk, and replacement with *Lactobacillus crispatus* intravaginal suppository probiotics reduced recurrent UTI in premenopausal women [93,94].

The female microbial community protects against bacterial vaginosis, which has been shown to result from replacing the microbiota rich in *Lactobacillus crispatus* with anaerobic species; thus, the urinary microbiota in healthy females differ significantly from those with bacterial vaginosis [91,95].

The urinary microbiota plays a role in preventing urge urinary incontinence (UUI) in females [86]. Both *Lactobacillus* spp. were cultured in females with and without UUI. In contrast, *Lactobacillus gasseri* was prevalent in the UUI cohort, and *Lactobacillus crispatus* was the prevalent species in the non-UUI females [86]. Also, *Gardnerella* was more frequently cultured in the urine specimen of the females with UUI than in the non-UUI cohort [86].

The urinary microbiota also reduces the risk of UTI in those with neurogenic bladder who showed a decrease in *Lactobacillus* and an increase in *Enterobacteriales*, indicating the possibility that this dysbiosis likely predisposed them to UTI [93].

The vaginal microbiome promotes healthy pregnancy outcomes, while *Escherichia coli* causes adverse events in pregnancy, including preterm birth [95,96]. Secretions from the female genital tract inhibit *Escherichia coli*. In health, the microbiota rich in *Lactobacillus crispatus* is associated with healthy pregnancy outcomes as it promotes *Escherichia coli* inhibitory activity of vaginal secretions, preventing its colonization and subsequently reducing the risk of preterm birth [95]. The *Lactobacillus* microbiota has also promoted fertility as its abundance in the female urogenital tract decreased in patients with infertility [97].

The microbial community in the urinary tract is important in preventing cellular rejection, as *Lactobacillus* in females and *Streptococcus* in males were reduced in those with interstitial fibrosis and tubular atrophy 6-8 months after renal transplantation. In contrast, lactobacillus normalized in females and streptococcus increased in males with excellent transplant function [86].

Vaginal microbiota transplantation (VMT) is an emerging therapeutic strategy aimed at restoring vaginal microbial balance, with the potential to improve the management of bacterial vaginosis and recurrent local infections, as well as to reduce the risk of sexually transmitted diseases and obstetric complications. However, further research is needed to establish donor selection criteria and assess long-term safety [90].

### 3.5. Central Nervous System

**Figure 2:**
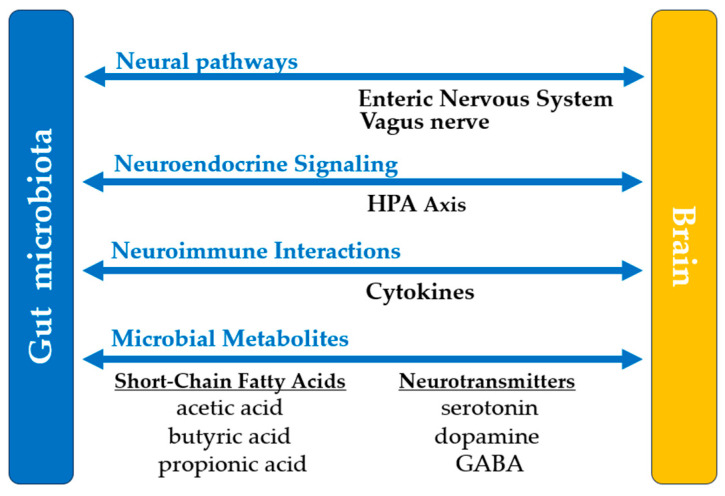
The gut–brain axis. HPA—hypothalamic–pituitary–adrenal; gamma-aminobutyric acid—GABA.

The gastrointestinal microbiome has emerged as a promising avenue for managing and improving neurological well-being. These microorganisms have been shown to influence the effects of neurological disorders through the gut–brain axis. This axis enables bidirectional communication between the central nervous system and gastrointestinal microflora through neural, endocrine, immune, and metabolic circuits, Figure 2. These interactions influence neuroprotection, blood–brain barrier permeability, cognitive function, motor skills, and overall brain health. Dysbiosis has been linked to various mental and neurological disorders. The recovery and symptom management of diseases like Alzheimer’s Disease (AD), Parkinson’s Disease (PD), autism spectrum disorder (ASD), and multiple sclerosis (MS) can be influenced by these microbial factions. In MS, dysbiosis is often characterized by a shift in the Firmicutes/Bacteroidetes ratio and an increased abundance of *Akkermansia muciniphila* [98]. While some studies have associated *Akkermansia* with mucin degradation and pro-inflammatory responses, more recent Mendelian randomization analyses indicate a causal protective effect of *Akkermansia muciniphila* against MS risk (OR 0.43, *p* = 1.37 × 10^−8^) [99]. This suggests that *Akkermansia*’s role may be context-dependent, influenced by host factors and overall microbial ecosystem composition.

The vagus nerve represents the primary communication pathway between the gut microbiota and the brain. Its intestinal receptors are indirectly stimulated by chemical substances produced by the microbiota, such as SCFAs, neurotransmitters, intestinal hormones [cholecystokinin and peptide YY(YY)], and mechanical stimuli (changes in pressure and intestinal wall distension) [100,101]. Many of these signals are mediated by enteroendocrine cells, which release signaling molecules that activate the afferent fibers of the vagus nerve. Microbial metabolites, such as SCFAs, are vital for optimal CNS functioning. SCFAs, such as acetate and butyrate, are pivotal in brain development, neurotransmitter synthesis, and neuroinflammation [100]. The gastrointestinal microbiota directly produces or regulates the production of neurotransmitters such as serotonin, dopamine, and gamma-aminobutyric acid (GABA).

In addition, gut microbes participate in the metabolism of tryptophan, an essential amino acid that serves as a precursor for serotonin, melatonin, and kynurenine. Several bacterial genera—including *Clostridium*, *Burkholderia*, *Pseudomonas*, *Streptomyces*, and *Bacillus*—are involved in transforming tryptophan into kynurenine and quinolinic acid, which deplete serotonin precursors and promote neuroinflammation. Conversely, other taxa such as *Holdemania*, *Ruminococcus*, and *Tyzzerella* favor the production of tryptamine and melatonin, supporting neurotransmitter synthesis and neuroprotection. The dysregulation of these pathways may contribute to neurological and psychiatric conditions, including depression, anxiety, and migraine, via impaired serotonin availability and altered pain perception [102].

The afferent component of the vagus nerve transmits the information received from the intestine to the nucleus of the solitary tract in the brainstem, where it is processed and relayed to brain regions involved in emotion regulation (amygdala and prefrontal cortex) and autonomic functions (hypothalamus). The hypothalamic–pituitary–adrenal (HPA) axis is modulated by signals transmitted from the gut through the vagus nerve, reducing stress and systemic inflammation. Studies have shown that probiotic strains such as *Lactobacillus* and *Bifidobacterium* elevate serotonin levels, aiding in the reduction in depressive and anxiety symptoms associated with AD and PD [100,101]. The production of dopamine, which is crucial for motor regulation, is influenced by gut microbes. Following probiotic treatment, an increase in dopamine signaling has been observed in PD patients [103].

The enteric nervous system (ENS), the “second brain”, consists of a complex autonomous neuronal network that plays a vital role in regulating gastrointestinal functions. It maintains close bidirectional communication with the central nervous system through the gut–brain axis. This communication involves the vagus nerve and neurotransmitters (serotonin, dopamine, and acetylcholine), influencing intestinal activity, emotion regulation, behavior, and autonomic responses. Moreover, the ENS plays a fundamental role in coordinating immune responses in the gut by interacting with immune cells, contributing to immunological tolerance and protection against pathogens. ENS dysfunctions can lead to the development of gastrointestinal and neuropsychiatric disorders.

Neurodegenerative diseases are aggravated by systemic inflammation, often caused by dysbiosis. Treatment with prebiotics and probiotics helps reduce inflammatory cytokine levels by restoring gastrointestinal microbial equilibrium. This mechanism has shown potential in conditions like MS, where lowering inflammation leads to a slowdown in disease progression [104]. The bacterial community of the stomach produces butyrate, which helps reduce inflammation by modulating immunity and supporting regulatory T cells [105]. In PD, FMT has demonstrated promise in reducing inflammatory markers [106]. In MS, inflammation markers declined in patients given SCFAs through prebiotics or supplemented with dietary fiber [103].

In AD, the gut microbial community can reduce amyloid-beta deposition by regulating systemic inflammation and oxidative stress. Studies have shown that prebiotic supplementation (which promotes the growth of *Bifidobacteria*) in individuals with AD is linked to enhanced memory and cognitive scores [107]. Dietary polyphenols broken down by gut bacteria can amplify neuroplasticity and cognitive resilience [108].

Gut dysbiosis is often evident in patients with PD. Supplementing them with *Lactobacillus plantarum* containing prebiotics has been linked to improved motor function. This effect is achieved by reducing alpha-synuclein buildup and boosting gut motility [103,109].

### 3.6. Cardiovascular System

Microbial metabolites affect the cardiovascular system, impacting vessels, vascular tone, lipids, and blood pressure. SCFAs produced from the microbial fermentation of dietary fibers in the colon are one of the main interactions between the microbiota and the cardiovascular system [110,111]. SCFAs, absorbed into the bloodstream, induce vasodilatory effects and contribute to regulating blood pressure. They interact with specific receptors on vascular smooth muscle cells, such as free fatty acid receptor (FFAR) 2 and FFAR3 [111,112]. The activation of these receptors promotes blood vessel dilation, thereby improving blood flow. Additionally, butyrate and acetate can stimulate the production of nitric oxide (NO), an essential mediator of vasodilation [113]. NO regulates vascular tone by activating soluble guanylate cyclase, increasing cyclic *guanosine* monophosphate levels, and activating protein kinase G, which leads to the relaxation of smooth muscle cells. NO also plays a significant role in endothelial protection and preventing thrombosis by inhibiting platelet aggregation and reducing the risk of atherosclerosis [114]. Moreover, SCFAs might modify the renin–angiotensin system, the key regulator of blood pressure. SCFAs can also reduce systemic inflammation, contributing to protection against cardiovascular diseases such as hypertension and atherosclerosis [111]. SCFAs can also exert opposing effects on blood pressure depending on the receptor activated. The activation of olfactory receptor 78 (Olfr78), expressed in renal afferent arterioles, has been shown to increase blood pressure by stimulating renin secretion [115]. The overall effect of SCFAs on blood pressure depends on the relative balance of these receptor pathways.

A second important microbial metabolite is TMA, which gut bacteria (*Clostridia*, *Escherichia*, *Desulfovibrio*, and *Anaerococcus*) make from dietary choline, phosphatidylcholine, and L-carnitine [28]. The production of TMA and its conversion to TMAO in the liver is associated with cardiovascular disease, but the production of TMAO is also regulated during normal, physiological states of lipid metabolism. The modulated cholesterol and lipid absorption in the intestine by the microbiota supports healthy blood lipid levels (essential to maintaining vascular integrity and function) [116].

Additionally, the gut–brain axis, the linkage of the gut microbiota with central nervous system signaling, also has implications for cardiovascular regulation. For instance, the vagus nerve may convey some effects of microbial metabolites on heart rate and autonomic control of the cardiovascular system. This neural pathway hints that the microbiota can affect the cardiovascular response indirectly through the modulation of heart rate variability as a measure of autonomic balance and cardiovascular fitness [117].

Recent research indicates that gut microbiota can influence salt sensitivity, a crucial factor in blood pressure regulation. The effect is mediated by gut microbial metabolites such as SCFAs and arachidonic acid [118].

### 3.7. Endocrine Function

The microbiota also heavily impacts the endocrine system, which governs hormone production and regulation. Several of these hormones, involved in metabolic and stress responses, are synthesized or regulated by gut bacteria. SCFAs synthesized by gut bacteria activate FFAR2 and 3 receptors on intestinal L cells, stimulating the secretion of glucagon-like peptide-1 (GLP-1 and PYY), which play a crucial role in glucose metabolism and appetite regulation [119].

The synthesis of ghrelin, a peptide secreted by X/A-like neuroendocrine cells in the gastric mucosa, can be indirectly stimulated by the presence of intestinal bacteria such as *Bifidobacterium*, *Lactobacillus*, or *Akkermansia* [116,120]. Ghrelin subsequently stimulates appetite and promotes the release of growth hormone. *Akkermansia* modulates the secretion of other key gut hormones, such as leptin, GLP-1, PYY, and cholecystokinin. It also increases satiety and improves metabolism [116,121].

In addition to that, the gut microbiota also interacts with the HPA axis, which comprises the core region for the body’s response to stress. The cortisol release that successively characterizes the HPA axis represents the primary regulator of the adaptive response to stress and of metabolism. Some gut bacteria, such as *Lactobacillus*, *Bifidobacterium*, and *Akkermansia muciniphila*, can activate this axis, affecting the release of the corticotropin-releasing hormone from the hypothalamus [122,123,124].

Microbial communities play an essential role in the metabolism of iodine, an essential component in thyroid hormone synthesis. Small intestine gut bacteria contribute to modulating iodine absorption to maintain normal thyroid gland functioning by producing thyroxine and triiodothyronine, which are responsible for regulating metabolism. Directly supporting endocrine balance and metabolic regulation, the efficiency with which microbes contribute to nutrient absorption, including iodine, indirectly [125].

The gut microbiota is known to influence both metabolic and steroid hormones, particularly estrogens and androgens. A key mechanism involves the production of enzymes, such as β-glucuronidase, by specific bacterial phyla, including *Bacteroidetes*, *Firmicutes*, *Verrucomicrobia*, and *Proteobacteria* [126]. These enzymes facilitate the deconjugation of estrogens in the gut, enabling their reabsorption into the bloodstream via enterohepatic recycling. This process is crucial in maintaining balanced circulating estrogen levels, essential for reproductive health, bone density, and cardiovascular function [127].

Bile acids’ potential to modulate the gut microbiome is essential for regulating the cardiovascular and endocrine systems. Bile acids derived from the liver are primary, while those altered by the bacteria found in the intestine are secondary forms. These secondary bile acids serve as ligands binding to the FXR and TGR5, which modulate glucose and lipid metabolism and energy expenditure. The right regulation of these pathways by the microbiota benefits metabolic health and the cardiovascular system [120].

### 3.8. Immune System

The human microbiota interacts with the immune system from the first moments of life, forming a bidirectional axis essential for maintaining a balance between the host and it. The immune system influences the composition of the microbiota, while the microbiota shapes the functions of innate and adaptive immunity.

Immediately after birth, the newborn begins to be colonized by a microbial flora that varies depending on environmental factors, nutrition, and the mode of delivery (vaginal or cesarean section). The microbiota tends to mature within the first three years of life, reaching a stable composition like an adult’s. During this “window of opportunity,” the dynamic relationship between the developing microbiota and the immune system plays a critical role in immune development.

At birth, newborns lack a fully developed immune system, making them vulnerable to microbial agents. The microbiota contributes to the maturation of T lymphocytes [Treg and T helper (Th1, Th2, and Th17)] and B lymphocytes, the synthesis of IgA (which plays a role in local defense) and serum IgG, and the inhibition of IgE production (involved in allergic reactions) [128,129]. Additionally, the microbiota regulates the development and activity of antigen-presenting cells (APCs), neutrophils, natural killer (NK) cells, and mast cells.

In germ-free animals, the absence of a microbiota results in structural lymphoid deficiencies and immunosuppression [128]. Immune homeostasis depends on the balance between pro-inflammatory responses (Th1, Th17) and anti-inflammatory responses (Treg and IgA). A diverse and balanced microbiota maintains this balance, producing metabolites such as SCFAs, essential for Treg regulation and the control of systemic inflammation.

The microbiota also contributes to the integrity of the epithelial barrier by stimulating mucus production, antimicrobial peptides (AMPs produced by Paneth cells), and strengthening intercellular junctions. Dysbiosis compromises this barrier, leading to bacterial translocation, the activation of inflammatory responses, and an increased risk of systemic inflammation.

Commensal microorganisms significantly induce local immune tolerance, reducing the risk of excessive inflammatory reactions. Polysaccharides synthesized by species such as *Bacteroides fragilis* modulate Th1/Th2 responses, promoting immune balance [128,129].

Dysbiosis caused by unbalanced diets, stress, or antibiotic treatments is associated with chronic inflammation and an increased risk of autoimmune diseases [129].

The human microbiota is pivotal in maintaining overall health and influences multiple physiological systems. Table 2 summarizes the effects of the human microbiota on various body systems.

### 3.9. Microbiota—A New Frontier in Diagnostic and Treatment

The individual composition of the microbiota presents opportunities for a personalized approach to various pathologies, offering diagnostic, treatment, and prevention solutions tailored to each patient. Genomic sequencing and metabolomic analysis allow for the detailed characterization of the individual microbiome and the identification of specific microbial biomarkers that guide clinicians toward the early detection of predisposition to or presence of pathologies such as metabolic syndrome, autoimmune diseases, or neurodegenerative conditions, Table 3.

In clinical practice, personalizing microbiota-based therapeutic strategies has proven effective in multiple fields, offering innovative solutions for microbial homeostasis regulation and improving patient health. Interventions include diet, probiotics, prebiotics, and postbiotics, as well as advanced strategies such as next-generation probiotics and various microbiota transplantation techniques. Probiotic-containing foods such as yogurt, kefir, and fermented vegetables, and prebiotic-rich sources like inulin-containing vegetables (e.g., garlic, onions, and asparagus) and whole grains contribute significantly to gut microbiota modulation. Targeted probiotics can stimulate beneficial bacterial species such as *Lactobacillus*, *Bifidobacterium*, and *Akkermansia*, reducing inflammation and improving epithelial barrier function. Prebiotics, substances that promote the growth of beneficial bacteria, are already being used to support metabolic and immune health. Postbiotics, active microbial metabolites, play an increasingly important role in maintaining microbial balance and treating inflammatory and metabolic diseases. A recent innovation is represented by next-generation probiotics, such as *Faecalibacterium prausnitzii* and *Akkermansia muciniphila*, which directly influence inflammation, intestinal homeostasis, and metabolism [129]. These species are studied extensively for therapeutic applications in obesity, diabetes, inflammatory bowel diseases, and neurodegenerative disorders. However, growing evidence highlights that short-term use of dietary, prebiotic, or probiotic interventions, typically under four weeks, only induces transient changes in the microbiota. A minimum intervention duration of three months is recommended to achieve stable and clinically relevant alterations in microbial composition and function [130].

Microbiota transplants have become promising therapeutic solutions for restoring microbial balance in severe conditions. FMT is an efficient treatment method, demonstrating success rates of over 90% in recurrent *Clostridioides difficile* infections, more than 60% in inflammatory bowel diseases, and less than 60% in extraintestinal conditions [131]. Other forms of transplantation, such as VMT, and SMT, are currently in experimental stages but show potential in treating dysbiosis related to gynecological, and dermatological conditions.

Moreover, the microbiota plays a key role in the body’s response to various therapies by metabolizing medications, influencing their bioavailability and efficacy. Personalized drug therapy, tailored to each patient’s individual microbiota profile, can prevent adverse reactions and increase therapeutic response rates.

Adapting a diet based on microbiota analysis can reduce the risk of developing and complicating certain metabolic diseases, such as obesity or diabetes mellitus.

Identifying the unique characteristics of the microbiota paves the way for precise, personalized therapies that can optimize health and improve quality of life.

The integration of these microbiota-based therapies opens new directions in personalized medicine, offering innovative solutions for chronic disease prevention and treatment, ultimately improving patients’ quality of life.

## 4. Conclusions

The microbiota represents a unique ecosystem essential for maintaining human health and is involved in multiple physiological functions, from metabolism to regulating immune responses and neuroprotection. It serves as the individual fingerprint of each human organism, enabling the development of personalized therapies, a promising solution for treating debilitating acute and chronic conditions such as infections, autoimmune diseases, neurodegenerative disorders, and metabolic syndromes. In-depth studies are required to understand the mechanisms through which the microbiota interacts with different bodily systems.

Understanding the role of the microbiota in regulating interactions between various organs and systems could open new perspectives in disease prevention through nutritional and pharmaceutical interventions. An integrated approach to microbiota research could significantly contribute to revolutionizing personalized medicine and improving patients’ quality of life.

## Figures and Tables

**Table 1:** Skin microbiota diversity.

Skin Region	Predominant Microorganisms	
Oily Zones	*Cutibacterium acnes* *Malassezia* spp.	*Staphylococcus* spp. *Corynebacterium* spp.
Moist Zones	*Staphylococcus* spp. *Corynebacterium* spp.	*Micrococcaceae* spp.
Dry Zones	*Staphylococcus epidermidis* *Micrococcus* spp. *Proteobacteria* spp.	*Bacteroidetes* spp. *Actinobacteria* spp.
Palms and Soles	*Corynebacterium* spp. *Staphylococcus* spp. *Malassezia* spp. *Aspergillus* spp.	*Cryptococcus* spp. *Rhodotorula* spp. *Epicoccum* spp.
Scalp	*Malassezia* spp. *Cutibacterium* spp.	*Demodex* spp.
Perianal Area	*Enterococcus* spp. *Escherichia coli*	*Bacteroides* spp.

spp.—species.

**Table 2:** The multisystem impact of microbiota.

System	Microbiota–System Relationship
1. Digestive System	Provides a protective barrier against pathogens. Supports digestion (polysaccharides and proteins). Enhances gut barrier integrity and reduces inflammation. Produces vitamins B-complex and K. Metabolizes dietary fibers Regulates glucose and lipid metabolism. Detoxifies xenobiotics and binds heavy metals. Influences systemic immune responses through modulation of gut-associated lymphoid tissue.
2. Integumentary System	Provides a protective barrier against pathogens. Regulates skin pH and supports lipid production. Stimulates skin regeneration and wound healing. Adapts to environmental factors. Modulates immune responses to maintain local homeostasis.
3. Respiratory System	Prevents colonization by respiratory pathogens. Modulates immune responses via alveolar macrophages and regulatory T cells. Supports mucosal barrier integrity and facilitates mucociliary clearance. Influences respiratory health through the gut–lung axis, producing SCFAs that modulate lung immunity. Contributes to IgA production, enhancing mucosal defenses.
4. Urinary System	Maintains urinary tract health by balancing microbial populations and preventing infections. Modulates local pH. Shares microbial communities with the reproductive system, supporting urogenital health.
5. Reproductive System	Regulates vaginal pH. Supports healthy pregnancy by modulating local immune responses. Protects against sexually transmitted infections through microbial competition and lactic acid production. Enhances fertility. Interacts with the urinary microbiota to maintain overall urogenital health.
6. Central Nervous System	Facilitates gut–brain communication via the vagus nerve. Produces neurotransmitters, influencing mood and cognition. Modulates stress responses. Reduces neuroinflammation. Promotes neuroprotection. Influences the progression and management of neurodegenerative diseases.
7. Cardiovascular System	Regulates blood pressure. Modulates lipid metabolism. Reduces systemic inflammation. Protects vascular integrity. Prevents thrombosis. Produces TMA, which is linked to cardiovascular health. Supports endothelial function and vascular tone.
8. Endocrine System	Regulating appetite and glucose metabolism. Modulates bile acid metabolism, influencing energy expenditure and lipid homeostasis. Affects cortisol production and stress adaptation. Supports thyroid hormone synthesis. Regulates estrogen levels.
9. Immune system	Influences the maturation and differentiation of T and B cells. Regulates the production of immunoglobulins. Balancs pro-inflammatory and anti-inflammatory responses. Modulates Treg activity and systemic inflammation. Promoting local immune tolerance. Reinforces the intestinal barrier. Prevents chronic inflammation and autoimmune diseases.

SCFAs—short-chain fatty acids; IgA—immunoglobulin A; TMA—trimethylamine; Treg—regulatory T cells.

**Table 3:** Microbial biomarkers and relevant bacteria across systems for diagnostic applications.

System	Relevant Microorganisms	Products/Biomarkers
1. Digestive System	*Faecalibacterium prausnitzii* *Akkermansia muciniphila* *Roseburia* spp. *Bacteroides* spp. *Lactobacillus* spp. *Eggerthella lenta* *Firmicutes* spp. *Firmicutes*/*Bacteroidetes* ratio	SCFAs mucin lipopolysaccharides GLP-1 PYY
2. Integumentary System	*Cutibacterium acnes* *Staphylococcus epidermidis* *Staphylococcus aureus* *Corynebacterium jeikeium* *Malassezia* spp. *Candida* spp. *Merkel cell polyomavirus*	free fatty acids AMPs lactic acid
3. Respiratory System	*Prevotella* spp. *Streptococcus* spp. *Veillonella* spp. *Haemophilus influenzae* *Moraxella catarrhalis*	IL-10 IL-6 IL-8
4. Urinary and Reproductive System	*Lactobacillus crispatus* *Lactobacillus gasseri* *Gardnerella vaginalis* *Corynebacterium* spp. *Escherichia coli*	
5. Central Nervous System	*Lactobacillus plantarum* *Bifidobacterium* spp.	SCFAs PYY serotonin dopamine GABA
6. Cardiovascular System	*Eubacterium coprostanoligenes* *Lactobacillus* spp. *Bifidobacterium* spp. *Ruminococcus* spp.	SCFAs TMA arachidonic acid
7. Endocrine System	*Akkermansia muciniphila* *Bacteroides* spp. *Bifidobacterium* spp.	SCFAs GLP-1 PYY β-glucuronidase leptin cholecystokinin
8. Immune System	*Bacteroides fragilis* *Lactobacillus* spp. *Bifidobacterium* spp. *Faecalibacterium prausnitzii*	SCFAs IL-10 AMPs

SCFAs—short-chain fatty acids; TMA—trimethylamine; GLP-1—glucagon-like peptide-1; PYY—peptide YY; AMPs—antimicrobial peptides; GABA—gamma-aminobutyric acid; IL — interleukin.

## Data Availability

No new data were created or analyzed in this study.
